# Application of Skimmed-Milk Flocculation Method for Wastewater Surveillance of COVID-19 in Kathmandu, Nepal

**DOI:** 10.3390/pathogens13050366

**Published:** 2024-04-29

**Authors:** Sarmila Tandukar, Ocean Thakali, Ananda Tiwari, Rakshya Baral, Bikash Malla, Eiji Haramoto, Jivan Shakya, Reshma Tuladhar, Dev Raj Joshi, Bhawana Sharma, Bhushan Raj Shrestha, Samendra P. Sherchan

**Affiliations:** 1 Organization for Public Health and Environmental Management, Lalitpur 44700, Nepal; sar1234tan@gmail.com (S.T.); othakali@gmail.com (O.T.); 2 Expert Microbiology Research Unit, Finnish Institute for Health and Welfare, 70701 Kuopio, Finland; ananda.tiwari@thl.fi; 3 Center of Research Excellence in Wastewater Based Epidemiology, Morgan State University, Baltimore, MD 21251, USA; rabar10@morgan.edu; 4 Interdisciplinary Center for River Basin Environment, University of Yamanashi, 4-3-11 Takeda, Kofu 400-8511, Yamanashi, Japaneharamoto@yamanashi.ac.jp (E.H.); 5 Central Department of Microbiology, Tribhuvan University, Kathmandu 46000, Nepal; 6 Environment and Public Health Organization, Kathmandu 44600, Nepal; 7 Department of Environmental Health, Tulane University, New Orleans, LA 70112, USA

**Keywords:** COVID-19, SARS-CoV-2, virus-concentrating method, wastewater-based epidemiology

## Abstract

Wastewater surveillance (WS) has been used globally as a complementary tool to monitor the spread of coronavirus disease 2019 (COVID-19) throughout the pandemic. However, a concern about the appropriateness of WS in low- and middle-income countries (LMICs) exists due to low sewer coverage and expensive viral concentration methods. In this study, influent wastewater samples (n = 63) collected from two wastewater treatment plants (WWTPs) of the Kathmandu Valley between March 2021 and February 2022 were concentrated using the economical skimmed-milk flocculation method (SMFM). The presence of severe acute respiratory syndrome coronavirus 2 (SARS-CoV-2) was tested by qPCR using assays that target the nucleocapsid (N) and envelope (E) genes. Overall, 84% (53/63) of the total samples were positive for SARS-CoV-2 according to at least one of the tested assays, with concentrations ranging from 3.5 to 8.3 log_10_ gene copies/L, indicating the effectiveness of the SMFM. No correlation was observed between the total number of COVID-19 cases and SARS-CoV-2 RNA concentrations in wastewater collected from the two WWTPs (*p* > 0.05). This finding cautions the prediction of future COVID-19 waves and the estimation of the number of COVID-19 cases based on wastewater concentration in settings with low sewer coverage by WWTPs. Future studies on WS in LMICs are recommended to be conducted by downscaling to sewer drainage, targeting a limited number of houses. Overall, this study supports the notion that SMFM can be an excellent economical virus-concentrating method for WS of COVID-19 in LMICs.

## 1. Introduction

Severe acute respiratory syndrome coronavirus 2 (SARS-CoV-2) can colonize the intestinal epithelial tissue of coronavirus disease-2019 (COVID-19)-infected individuals and is excreted in feces, even beyond the period of its detection in nasal swab sampling, a gold standard method for clinical testing [1]. This indicates that wastewater monitoring can be an alternative surveillance modality at the population level. Several studies have established the utility of wastewater surveillance (WS) in COVID-19 epidemiology [2,3,4]. Medema et al., 2020, first reported the presence of SARS-CoV-2 in the wastewater of several Dutch cities concurrent with the week the first case was reported [3]. In Australia, SARS-CoV-2 RNA was quantified in wastewater and the number of COVID-19-infected persons within the wastewater catchment area was estimated based on the concentration of SARS-CoV-2 RNA in the wastewater [2]. The majority of such WS studies on COVID-19 have been carried out in developed, high-income countries with access to resources and proper sewer networks, covering a known population size in a given wastewater treatment plant (WWTP) catchment area, making it fairly easier to perform WS. Conversely, the implementation of WS in developing countries poses significant challenges and deserves further attention.

Considerable efforts are underway in developing sampling strategies and economical virus-concentrating methods to promote WS in low- and middle-income countries (LMICs). LMICs often suffer from limited clinical diagnostic tools, and WS can provide added benefits of determining the prevalence of COVID-19 as well as helping to identify emerging hotspots. A study in Quito, Ecuador, detected SARS-CoV-2 RNA using an economical skimmed milk flocculation method (SMFM) to concentrate viral particles in river water samples collected from an urban river impacted by urban wastewater as an alternative to influent from a WWTP [5]. Similar to the situation in Quito, the treatment and management of wastewater in the densely populated Kathmandu Valley, Nepal, is poorly practiced, with only one functioning WWTP. The citywide inclusive sanitation concept in the Kathmandu Valley is still poorly developed. Theoretically, there are five WWTP to treat municipal wastewater within the Valley. Nevertheless, only one WWTP is currently operating, with the ability to treat ~30.6 MLD [6]. This is considerably low compared to the wastewater generated within the valley (−200 MLD) [7]. The final destination for all the human waste, including feces, is either a wastewater treatment plant or a nearby river. Limited numbers of houses are covered by the WWTP and the sewage from the rest is emptied into rivers without treatment, enabling the detection of SARS-CoV-2 RNA in river water, as reported previously [4]. The first study that conducted WS of COVID-19 in Nepal utilized filtration through an electronegative filter membrane, followed by ultrafiltration, as primary and secondary virus concentrating methods, respectively [4]. These methods require expensive filter setups and ultrafiltration devices which can be fairly impractical for long-term surveillance in LMICs. In the clinics, we believe that inadequate laboratory facilities and materials, high costs, and lack of qualified personnel to conduct clinical testing have limited mass testing in Nepal. Furthermore, a high proportion of asymptomatic cases might have also undermined the actual extent of SARS-CoV-2 spread in the Valley.

Based on this background, this study aimed to detect SARS-CoV-2 RNA in wastewater using the economical SMFM method and to assess the relationship between the concentration of SARS-CoV-2 RNA in wastewater collected from WWTPs and the number of COVID-19 cases in the Kathmandu Valley, Nepal. To the best of our knowledge, this is the first study to concentrate SARS-CoV-2 RNA from WWTPs using SMFM in Kathmandu, Nepal.

## 2. Materials and Methods

### 2.1. Sample Collection and Virus Concentration

A total of 63 grab influent wastewater samples were collected from the centralized Guheswori WWTP (GHTP; n = 35) and the decentralized Gokarna WWTP (GKTP; n = 28) in the Kathmandu Valley, Nepal, from March 2021 to February 2022 (Table 1). From March through June, sampling was performed once a month; in July, it was performed twice a month; and in following months, samples were taken weekly from each sampling location between 8 and 10 am. The various government policies in effect during the sampling period caused the initial disparity in the frequency of sampling. Three different volumes of wastewater were collected: two 500 mL samples (one for sample concentration and one for chemical parameter analysis for future purposes) and a third sample of 100 mL (for archiving, stored at −20 °C) from both WWTPs. Autoclaved Nalgene bottles were used and sanitized externally with virucidal disposable wipes prior to transportation. On each collection day, the research assistant collected the samples and transported them in an icebox to the laboratory as soon as possible (within 1 h). Samples were processed for primary concentration on the same day. In situations where this was not possible, wastewater samples were stored at 4 °C and processed the next day. GHTP is based on an activated sludge system designed to treat 32.4 million liters per day (MLD) (Kathmandu Upatyaka Khanepani Limited, Kathmandu, Nepal, 2019). In contrast, GKTP is currently not functioning and directly drains wastewater into the Bagmati River. The concentration of the virus in wastewater was established using the SMFM. Briefly, 2 mL of 5% skimmed milk solution was added to 200 mL of wastewater and 0.5 M HCl was added to adjust the pH to 3.0–4.0. The mixture was then agitated at 200 rpm for 2 h at room temperature (20–25 °C) on a horizontal table shaker to allow flocculation. The flocculation was allowed to sediment by letting it sit at room temperature, and it was then transferred into 50 mL Falcon tubes for centrifugation at 3500× *g* for 30 min at 4 °C. The supernatant was discarded carefully without disturbing the pellet. The pellet was resuspended in 5 mL sterile phosphate-buffered saline (PBS) (pH 7.4) by vortexing at maximum speed for 10 min. The final viral concentrate was recovered and stored at −80 °C until further processing.

### 2.2. RNA Extraction and qPCR

One hundred and forty microliters of the virus concentrates were used to extract viral RNA, using a QIA-amp Viral RNA Mini Kit (Qiagen, Hilden, Germany) following the manufacturer’s instructions. RT-qPCR was performed on a QuantStudio5 thermal cycler (Applied Biosystems, Foster City, CA, USA) and all samples were tested in duplicates. N and E gene assays specific for SARS-CoV-2 were tested using a XABT Multiple Real-Time PCR Kit (Beijing Applied Biological Technologies Co., Ltd., Beijing, China). The RT-qPCR reaction mixture (20 μL) comprised 10 μLTaqMan™ Gene Expression Master Mix (Applied biosystem), 5 μL RNA template, 2 μL nuclease-free water, 1 μL 20× reverse transcriptase, and 2 μL primer/probe mix for N or E genes. Thermal conditions for RT-qPCR were 45 °C for 10 min for RT, followed by 95 °C for 5 min, 45 cycles of 95 °C for 15 s, and 60 °C for 45 s. The negative control comprised of PCR-grade water. The standard plasmid of SARS-CoV-2, obtained from IDT (Coralville, IA, USA), was used as a positive control and to prepare standard curves with concentrations ranging from 10^1^ to 10^6^ copies/reaction.

### 2.3. Statistical Analysis

Microsoft Excel 2019 was used for all statistical analyses (Microsoft Corporation, Redmond, WA, USA) and the preparation of tables. A *p* value of less than 0.05 was deemed significant. Non-detects were considered negative for this study. Limit of detection, defined here as the lowest number of copies that was amplified for N and E genes, was found to be 3.5 log_10_ copies/L. The relationship between the concentration of SARS-CoV-2 RNA in raw wastewater samples and the number of confirmed cases of COVID-19 in Kathmandu (where WWTP is located) was determined by calculating Pearson coefficients.

## 3. Results and Discussion

### 3.1. Detection of SARS-CoV-2 RNA in Wastewater Samples

Overall, 84% (53/63) of the total samples were positive for SARS-CoV-2 using at least one of the tested genes, indicating the effectiveness of SMFM for the concentration of SARS-CoV-2 from wastewater. This finding is in agreement with a previous study that validated SMFM for the effective recovery of SARS-CoV-2 RNA from wastewater [8]. SMFM is based on the principle that viruses adsorb to pre-flocculated skimmed milk proteins, facilitating the settling of viral particles in pellets under minimal centrifugal force. During the initiation of this study, economical methods that had been validated to concentrate viruses from wastewater were limited to polyethylene glycol (PEG) precipitation and ultracentrifugation [9]. Nevertheless, ultracentrifugation under high speeds of >100,000× *g* requires expensive ultracentrifuge devices, the use of which is not always feasible in LMICs such as Nepal. PEG precipitation is a commonly used method to concentrate viruses in wastewater, whereas a minimal number of studies have conducted WS of SARS-CoV-2 in LMICs using SMFM [5,10,11]. Our study supports the notion that SMFM can be an excellent method for WS of COVID-19 in LMICs.

The concentration of SARS-CoV-2 RNA in this study was found to be higher than the previous study that first quantified the concentration of SARS-CoV-2 RNA in raw wastewater of the same region [4]. The first study was conducted between July 2020 and February 2021, coinciding with the early onset of the COVID-19 pandemic. In this study, daily cases with a maximum of 11,500 were documented from March 2021 to February 2022, compared to the maximum of 4500 instances recorded during the previous study. The emergence of various variants of SARS-CoV-2, resulting in a high number of COVID-19 infections, and the possible transition of COVID-19 from a pandemic to an endemic state, may account for the discrepancy in RNA concentration between the two studies. One shortcoming of this study is the failure to determine the circulating variants of SARS-CoV-2 during the study period. As the viral concentrate and RNA has been archived, future studies will be conducted to demonstrate the temporal variation in circulating variants of SARS-CoV-2 in Kathmandu, Nepal.

As shown in Table 2, SARS-CoV-2 RNA was detected in 79% (50/63) and 78% (49/63) of the total samples via qPCR by N and E gene assays, respectively. In contrast to previously conducted studies [12,13], no significant difference in concentrations of SARS-CoV-2 RNA quantified with different qPCR assays was noted in this study (paired t-test, *p* > 0.05). Quantification bias can arise from the diminished sensitivity of qPCR assays due to nucleotide mismatches between the viral genome and primers and probe. The use of two or more assays for WS of SARS-CoV-2 is common and we strongly recommend it to improve the sensitivity and accuracy of WS. In this study, samples collected from GHTP showed a high positivity rate of 97%, whereas samples of GKTP showed a lower prevalence of 68% only. GKTP is a decentralized treatment plant that serves a small population. In contrast, GHTP typically serves larger populations compared to GKTP, and the difference in the positive rate could be due to the possibility of higher numbers of COVID-19-infected individuals in areas served by GHTP. Here, wastewater samples were analyzed using qPCR, despite the fact that digital PCR (dPCR) is least likely to be affected by potential PCR inhibitors present in wastewater compared to qPCR [14]. dPCR is expensive and is nearly impossible to use for long-term WS in LMICs.

### 3.2. Association between COVID-19 Cases and SARS-CoV-2 RNA Concentration in Wastewater

Pearson’s correlation coefficient test was performed on the wastewater concentration of SARS-CoV-2 and the total number of active COVID-19 cases reported on the same day in the Kathmandu Valley. The Ministry of Health, Government of Nepal’s COVID-19 dashboard provided information on the number of active cases reported. No correlation was observed between the wastewater concentration of SARS-CoV-2 in either WWTP based on both N and E gene assays and the number of active COVID-19 cases reported on the same day in Kathmandu (*p* > 0.05) (Table 3). Similar findings of no correlation between fecal-marker-normalized SARS-CoV-2 RNA concentration and the number of active cases have been reported previously in the same study area [4]. The limitations of the grab sampling approach, the low number of houses covered by the WWTP in the valley, the possible decay of SARS-CoV-2 RNA in sewers, and underreporting of the number of active cases reported by the government in the valley due to a lack of clinical testing and stigma associated with COVID-19 at the beginning of the COVID-19 pandemic can explain why no correlation was seen in our study. Our correlation result also cautions against predicting future COVID-19 waves and the estimation of the number of COVID-19 cases based on wastewater concentration in settings with lower sewer coverage by WWTPs. Napit et al., 2023, conducted WS of SARS-CoV-2 in the same study area by collecting wastewater from sewers because of the possible underrepresentation of citywide wastewater by wastewater samples collected from the WWTP only [15]. It is generally recommended to downscale WS to a smaller area by sampling sewer lines corresponding to the area of interest to improve the accuracy of WS in LMICs where coverage of WWTPs is inadequate [16].

## 4. Conclusions

In this study, 84% (53/63) of the total samples were positive for SARS-CoV-2 by at least one of the tested qPCR assays. No correlation was observed between the number of active COVID-19 cases and SARS-CoV-2 RNA concentrations in wastewater collected from the two WWTPs (*p* > 0.05). Future studies on WS in LMICs are recommended to be conducted by downscaling to sewer drainage targeting a limited number of houses using an economical virus concentration method with a quick turnover time. Overall, this study supports the notion that SMFM can be an excellent economical virus-concentrating method for WS of COVID-19 in LMICs.

## Figures and Tables

**Table 1:** Description of sampling sites.

Site ID	Facility Name	Metropolitan/Municipality	Site Latitude	Site Longitude	Number of Collected Samples (n = 63)
GHTP	Guheswori WWTP	Kathmandu	27.711844	85.355212	35
GKTP	Gokarna WWTP	Gokarna	27.740016	85.390234	28

**Table 2:** Detection of N and E genes of SARS-CoV-2 in wastewater.

Sample Site	No. of Samples	N Gene		E Gene		No. of Samples Positive for at Least One Assay (%)
		No. of Positive Samples (%)	Concentration (Range; log_10_ copies/L)	No. of Positive Samples (%)	Concentration (Range; log_10_ copies/L)	
GHTP	35	34 (97)	4.0–8.3	32 (91)	4.0–7.4	34 (97)
GKTP	28	16 (57)	4.0–7.0	17 (61)	3.5–7.0	19 (68)
Total	63	50 (79)	4.0–8.3	49 (78)	3.5–7.4	53 (84)

**Table 3:** Relationships in the concentrations between SARS-CoV-2 detected from GKTP and GHTP and number of active cases reported.

Sampling Site	R Values ^$^	
	N Gene	E Gene
GKTP	−0.12	−0.11
GHTP	0.03	0.17

^$^ Statistically not significant (*p* > 0.05).

## Data Availability

Data are available on request.
